# Long-Term Clinical Outcomes of Wedge-Shaped Implants Inserted in Narrow Ridges: A 7-Year Follow-Up Multicenter Prospective Single-Arm Cohort Study

**DOI:** 10.3390/jcm14176299

**Published:** 2025-09-06

**Authors:** Antonio Rapani, Tomaso Vercellotti, Claudio Stacchi, Gianluca Gregorig, Francesco Oreglia, Emanuele Morella, Teresa Lombardi

**Affiliations:** 1 Department of Medical, Surgical and Health Sciences, University of Trieste, 34129 Trieste, Italy; claudio@stacchi.it; 2 Department of Surgical Sciences and Integrated Diagnostics, University of Genova, 16100 Genova, Italy; tomaso@vercellotti.com; 3 Private Practice, 33012 Sappada (UD), Italy; gregoriggl@libero.it; 4 Private Practice, 37126 Verona, Italy; foreglia@yahoo.it; 5 Private Practice, 20099 Sesto San Giovanni (MI), Italy; info@studiodrmorella.com; 6 Department of Medical and Surgical Specialties, Radiological Sciences and Public Health, University of Brescia, 25123 Brescia, Italy; drteresalombardi@libero.it

**Keywords:** wedge implant, narrow ridge, piezosurgery, atrophic ridge, minimally invasive, patient-related outcomes

## Abstract

**Background:** Wedge-shaped implants have been proposed as a minimally invasive solution for narrow alveolar ridges, aiming to avoid bone augmentation. While the short-term results are promising, long-term clinical evidence remains limited. **Methods:** This multicenter prospective single-arm cohort study reports the 7-year outcomes of tissue-level wedge-shaped implants (1.8 mm thickness) placed without grafting in horizontally atrophic ridges (mean thickness 3.73 ± 0.36 mm). Clinical and radiographic evaluations were performed on 45 implants (34 patients). **Results:** At the 7-year post-loading follow-up, the implant survival rate was 95.5%, with two failures recorded—one early loss and one due to peri-implantitis. Peri-implant mucositis was observed in 5 implants (11.4%), while peri-implantitis was diagnosed in 3 implants (6.8%). No mechanical complications were reported. The mean marginal bone loss (MBL) was 1.45 ± 1.41 mm, measured relative to the implant shoulder. Multivariate linear regression identified older age (β = +0.040; *p* = 0.012) and mandibular implant placement (β = +1.39; *p* = 0.007) as significant predictors of greater bone loss. **Conclusions:** Wedge-shaped implants demonstrated high long-term survival and stable marginal bone levels in narrow ridges without the need for bone augmentation. Age and mandibular location negatively influenced long-term bone stability, while smoking, gender, and history of periodontitis were not significant predictors.

## 1. Introduction

Over the past few decades, implant dentistry has evolved toward less invasive protocols and shorter treatment times, particularly in anatomically challenging situations such as alveolar ridge atrophy following tooth extraction, which often leads to horizontal and/or vertical bone deficiencies [1]. Despite these advances, the rehabilitation of horizontally atrophic ridges often still require bone augmentation procedures to achieve sufficient volume for standard implant placement [2]. Several augmentation techniques are available and have proven to be effective in increasing bone volume [3,4,5,6,7]; however, they tend to be invasive, costly, and time-consuming, requiring specific surgical expertise and potentially impacting patient acceptance and quality of life [8]. To overcome these limitations, narrow-diameter implants have been proposed as a conservative alternative to reduce the need for horizontal augmentation. While beneficial in specific scenarios, their indications remain limited, as factors such as biomechanics, bone quality, and prosthetic load must be carefully considered before use [9,10].

In recent years, wedge-shaped implants were introduced as an alternative treatment modality to address these limitations. Inspired by the historical design of blade implants [11], wedge-shaped implants are characterized by a narrow bucco-lingual profile and flat lateral surfaces, designed to engage both buccal and lingual cortices, optimizing stability and bone-to-implant contact in narrow ridges. Moreover, compared to traditional blade implant protocols, wedge implants benefit from modern advancements in implant site preparation techniques [12,13], surface treatments [14], and prosthetic workflows [15]. Previous studies have demonstrated the feasibility of this approach, reporting positive short-term clinical outcomes in the treatment of both partial and total edentulism with wedge implant-supported fixed solutions [16,17,18,19]. However, despite these promising findings, long-term data are necessary for evaluating the durability and predictability of this innovative implant design. Parameters such as implant survival rate and peri-implant health conditions should be carefully assessed over time to understand the behavior of these implants and their reliability across different patients and clinical scenarios. Furthermore, peri-implant marginal bone stability is recognized as a key indicator of implant health and long-term favorable prognosis [20,21]. Marginal bone loss (MBL), a multifactorial process, can be influenced by various factors including implant geometry, surgical technique, prosthetic connection, and patient-specific variables [22]. Given the unconventional geometry of wedge-shaped implants and their unique bone engagement strategy, monitoring crestal bone changes over time is of particular interest.

The aim of this multicenter prospective study was to provide long-term clinical data on wedge-shaped dental implants by evaluating key outcomes, including implant survival, peri-implant tissue health, and marginal bone level stability. This paper presents the 7-year post-loading results collected from six clinical centers.

## 2. Materials and Methods

### 2.1. Study Design and Patient Selection

This prospective multicenter single-arm cohort study reports the 7-year follow-up of a population previously investigated in a short-term study evaluating wedge-shaped implants placed in horizontally atrophic ridges without bone augmentation [16]. The earlier publication presented data at 1-year post-loading, whereas the present study extends the observation to assess long-term clinical and radiographic outcomes. Surgical and prosthetic treatments were performed between January 2017 and July 2018 across six private clinical centers in Italy (T.V., F.O., T.L., G.G., E.M., C.S.) and involved 44 patients who received a total of 59 implants. Detailed inclusion and exclusion criteria, as well as surgical and prosthetic protocols, were described in the 1-year follow-up publication [16]. The present follow-up was conducted in the same patient cohort and clinical settings to maintain methodological consistency. All clinical and radiographic assessments were performed by the same investigators involved in the previous study. This study was carried out in accordance with the Declaration of Helsinki and received ethical approval from the Regional Ethics Committee of Calabria, Sezione Area Nord (No. 1/2017), and it was registered in a public clinical trials database (www.clinicaltrials.gov—NCT03290729, last accessed on 1 August 2025). Written informed consent was obtained from all participants at both initial enrollment and the 7-year follow-up examination.

### 2.2. Implant Characteristics and Surgical Protocol

**Figure 1:**
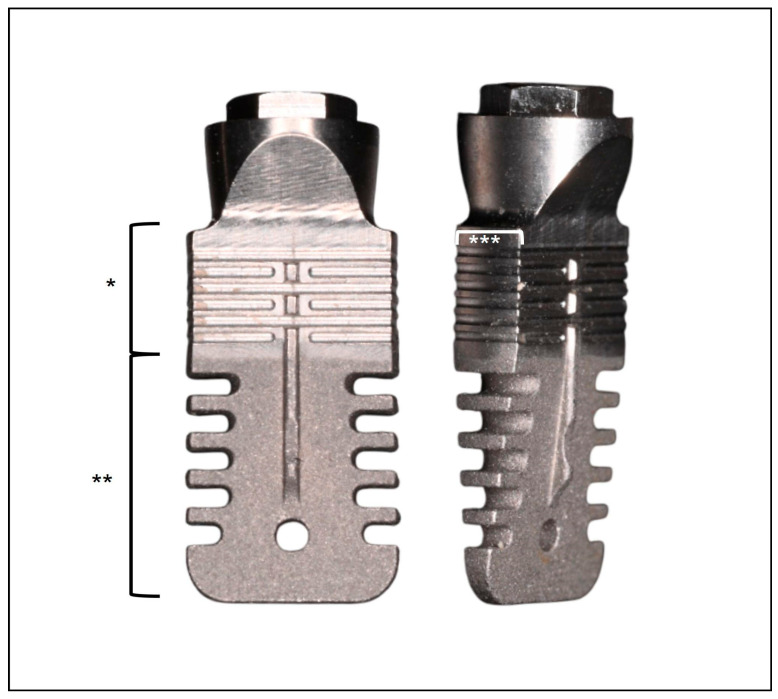
Investigational device: a tissue-level, wedge-shaped dental implant with an external connection. *: minimally rough surface; **: moderately rough surface; ***: 1.8 mm thickness.

The implants evaluated in this study were commercially available, tissue-level, wedge-shaped titanium implants (Rex TL, Rex Implants, Columbus, OH, USA), featuring a mesio-distal width of 5 mm and a bucco-lingual width of 1.8 mm, and characterized by macro-grooves at 0.5 mm intervals along the middle and apical portions of the mesial and distal surfaces (Figure 1). The implant surface had a hybrid treatment: a minimally rough coronal portion and a moderately rough middle and apical region. Implant lengths (9, 11, 13, or 15 mm) were selected based on available bone height and operator preference. Implant site preparation was performed exclusively using piezoelectric instrumentation (Piezosurgery Touch, Mectron, Italy), following a standardized protocol provided by the manufacturer. Implants were then press-fitted into the osteotomy using a dedicated magnetic mallet (IPD, Rex Implants, Columbus, OH, USA). All surgeries were performed under local anesthesia with preoperative antibiotic prophylaxis. Healing was non-submerged, and prosthetic loading occurred after a healing period of six months. Following prosthetic delivery, all patients were enrolled in a regular maintenance program and recalled every four months for professional hygiene and monitoring of periodontal and peri-implant conditions throughout the entire study period.

### 2.3. Clinical and Radiographic Evaluation

**Figure 2:**
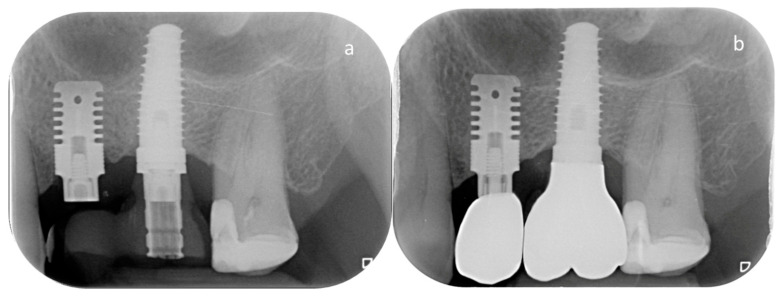
Periapical radiographs taken at baseline (T0) (**a**) and after 7 years (**b**) to evaluate marginal bone changes.

At the 7-year follow-up (T7), each implant underwent a comprehensive clinical and radiographic evaluation to assess its functional status (Figure 2). Parameters included implant mobility, pain on percussion or palpation, and peri-implant probing to evaluate probing depths and bleeding and/or exudate on probing. Digital periapical radiographs were taken using a standardized long-cone technique with the same exposure settings used in previous assessments to minimize variability (65–90 kV, 7.5–10 mA and 0.22–0.25 s). Radiographic analysis was performed by an independent, blinded examiner (A.R.), who had also conducted the measurements in the previously published short-term study on the same cohort, to ensure consistency and objectivity across assessments. The evaluation of marginal bone levels was conducted on both mesial and distal implant aspects, measuring the vertical distance between the implant shoulder and the most coronal point of bone-to-implant contact. A positive value was assigned when the bone crest was located coronal to the implant shoulder, and a negative value when it was apical. Measurements were calibrated based on the actual implant length and diameter and carried out using ImageJ software, version 1.51 (NIH, Bethesda, MD, USA). Examiner calibration was performed by comparing ten randomly selected radiographs against a reference examiner (C.S.). The intra- and inter-examiner agreement for linear measurements within ±0.1 mm was 93.4% and 87.6%, respectively, indicating high measurement reliability.

### 2.4. Outcome Measures

The primary outcome of the present study was implant survival, defined as the presence of the implant *in situ* without removal for any reason at the 7-year recall.

Secondary outcomes included:

- Marginal bone loss (MBL) at T7. MBL was assessed radiographically on both mesial and distal aspects of each implant and expressed as the mean value in millimeters.
- Biological complications (peri-implant mucositis or peri-implantitis) at T7. Mucositis and peri-implantitis were diagnosed according to the criteria established by Workgroup 4 of the 2017 World Workshop on the Classification of Periodontal and Peri-Implant Diseases and Conditions [23]. Peri-implant mucositis is defined by the presence of bleeding and/or suppuration on gentle probing, with signs of soft tissue inflammation and no radiographic bone loss beyond initial remodeling. The diagnostic criteria for peri-implantitis included: (1) presence of bleeding and/or suppuration on gentle probing; (2) increased probing depth relative to baseline measurements; and (3) radiographic evidence of marginal bone loss ≥0.5 mm compared to the baseline radiograph.
- Mechanical complications, including screw loosening or fracture, implant fracture, prosthetic component fracture or chipping of veneering material.

All outcome measures were analyzed descriptively, and no changes were made to the evaluation protocol compared to the original 1-year study.

### 2.5. Statistical Analysis

Descriptive statistics were used to summarize patient demographics, implant distribution, and outcome measures. Continuous variables were reported as mean ± standard deviation (SD) or median with interquartile range (IQR), depending on data distribution. Categorical variables were expressed as absolute frequencies and percentages.

Implant survival was estimated using the Kaplan–Meier method, with implant loss as the event and drop-outs censored at the last available follow-up.

Marginal bone level (MBL) was assessed at four timepoints: baseline at implant insertion (T0), 6 months post-insertion at prosthesis delivery (T1), 12 months after loading (T2), and 7 years after loading (T7). The first three timepoints (T0–T2) were derived from the one-year follow-up study published in 2020 [16], while T7 represents the newly acquired long-term data. Normality of the distribution of the MBL variations between T0 and T7 (T7–T0) was evaluated using the Shapiro–Wilk test. As normality was confirmed, a paired *t*-test was used to assess the statistical significance of longitudinal changes between baseline and 7-year follow-up.

To identify factors associated with long-term crestal bone changes, a multivariate linear regression analysis was conducted using T7–T0 as the dependent variable. Independent variables included age, gender, smoking status, history of periodontitis, baseline bone width (T0), and implant site (maxilla vs. mandible). Model assumptions were verified by inspection of residuals and homoscedasticity. Cluster-robust standard errors (patient-level) were applied to the regression estimates to account for the non-independence of multiple implants within the same patient.

All statistical analyses were performed using Python software (Python Software Foundation. Python Language Reference, version 3.11. Available at: https://www.python.org, last accessed on 24 July 2025). The level of statistical significance was set at α = 0.05.

## 3. Results

### 3.1. Study Population

At baseline (T0), 59 wedge-shaped implants were placed in 44 patients. At the 7-year follow-up, 45 implants in 34 patients were available for evaluation. During the follow-up period, 10 patients (14 implants) dropped out of this study: five patients (9 implants) were deceased, while the remaining five (5 implants) discontinued the scheduled maintenance program or failed to attend the 7-year recall visit. The final study cohort included 21 females and 13 males, with a mean age at implant placement of 59.6 ± 11.3 years. Among them, 9 were smokers and 25 non-smokers. Fourteen patients had a history of treated periodontitis, while 20 had no prior periodontal disease.

All implants assessed at 7 years had been placed in horizontally atrophic ridges without any bone grafting or augmentation procedures performed either prior to or concurrently with implant placement. Implant lengths included 15 implants of 9 mm and 30 implants of 11 mm. Anatomically, 12 implants were placed in the maxilla and 33 in the mandible. The mean ridge width at baseline, measured 1 mm apical to the crest, was 3.73 ± 0.36 mm. Demographic and clinical characteristics are summarized in Table 1.

### 3.2. Clinical Outcomes

At T7, the implant survival rate was 95.5% (43 surviving implants out of 45). One implant was lost within the first month after placement, and the second failed after six years of functional loading due to peri-implantitis. When analyzed with the Kaplan–Meier method including all 59 implants placed at baseline, the estimated 7-year survival probability was 95.6% (95% CI: 83.4–98.9%).

According to the diagnostic criteria established by Workgroup 4 of the 2017 World Workshop on the Classification of Periodontal and Peri-Implant Diseases and Conditions [23], 5 implants (11.4%) exhibited peri-implant mucositis, and 3 implants (6.8%) were diagnosed with peri-implantitis at the 7-year follow-up. Additionally, one implant had been lost prior to T7 due to peri-implantitis. Therefore, the cumulative prevalence of peri-implantitis over the 7-year period was 4 out of 44 implants (9.1%). No mechanical complications—including screw loosening or fracture, implant fracture, prosthetic component fracture or chipping of veneering material—were recorded.

**Figure 3:**
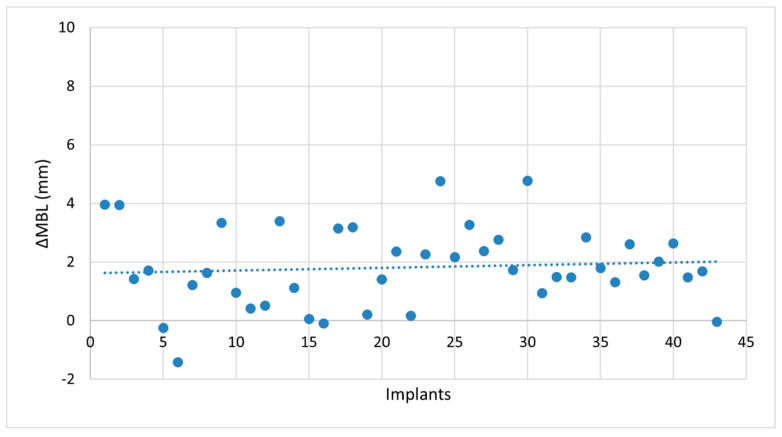
Scatter plot of ΔMBL at 7 years across all implants (total = 43). The dashed line indicates the mean value.

No statistically significant differences were detected between mesial and distal MBL measurements at any timepoint. Therefore, a single mean MBL value was calculated per implant and used for subsequent analyses. Radiographic evaluation demonstrated progressive peri-implant marginal bone resorption over the 7-year follow-up period. At baseline (T0), implants were placed slightly subcrestally, with a mean distance of 0.37 ± 0.59 mm between the implant shoulder and the crestal bone. At T7, the mean marginal bone level (MBL) was 1.45 ± 1.41 mm apical to the implant shoulder. The mean change in MBL (ΔMBL) from T0 to T7 was 1.82 ± 1.37 mm, which includes 0.37 ± 0.59 mm of bone remodeling (due to subcrestal placement) and 1.45 ± 1.41 mm of marginal bone loss [24]. Normality of the paired differences was confirmed using the Shapiro–Wilk test (*p* = 0.860). A paired *t*-test demonstrated a significant difference between baseline and 7-year measurements (t = 8.74; *p* = 0.00012). The distribution of ΔMBL values across all implants is shown in Figure 3.

To investigate potential predictors of long-term bone resorption, a multivariate linear regression model was built using ΔMBL as the dependent variable. The model included patient age, gender, smoking status, history of periodontitis, baseline bone width (T0), and implant site (maxilla vs. mandible) as covariates. Among these, two variables reached statistical significance, age (β = +0.040; *p* = 0.012) and jaw location, with mandibular implants showing significantly higher bone loss compared to maxillary ones (β = +1.39; *p* = 0.007).

Other factors, including gender (*p* = 0.280), smoking (*p* = 0.948), history of periodontitis (*p* = 0.612), and bone width at T0 (*p* = 0.957), were not significantly associated with bone loss. The model demonstrated an adjusted R^2^ of 0.173, indicating that approximately 17% of the variability in long-term MBL changes could be explained by the included predictors.

A detailed summary of regression coefficients and confidence intervals is provided in Table 2.

## 4. Discussion

The present 7-year multicenter prospective single-arm cohort study evaluated the long-term clinical performance of wedge-shaped implants for the rehabilitation of narrow alveolar ridges (mean width 3.73 ± 0.36 mm) without the need for bone augmentation. The findings demonstrate a high implant survival rate (95.5%) and overall favorable clinical outcomes, supporting the use of this implant design as a minimally invasive treatment option for horizontally atrophic ridges. The cumulative 7-year implant survival rate of 95.5% is comparable to that reported in long-term clinical studies and systematic reviews on conventional implants placed in more favorable bone conditions [25,26,27,28]. However, this should not be interpreted as evidence of equivalence, as the clinical scenario differs substantially: in ridges with a mean width of ~3.7 mm, conventional implants cannot be placed without simultaneous or staged horizontal bone augmentation. Thus, wedge-shaped implants offer comparable long-term survival while avoiding grafting procedures in situations where standard implants would typically require augmentation.

One early failure occurred within the first month, likely related to non-osseointegration, and one late failure was attributed to peri-implantitis. Importantly, the cumulative prevalence of peri-implantitis was 9.1%, which includes three implants diagnosed at T7 and one implant lost during the observation period. Peri-implant mucositis was observed in 11.4% of the implants. These values fall within, and in some cases below, the ranges reported in systematic reviews for standard implants [29,30,31], suggesting that the unconventional design of wedge-shaped implants does not appear to increase the risk of biological complications over the long term.

Radiographic evaluation showed a mean marginal bone loss (MBL) of 1.45 ± 1.41 mm after 7 years, measured relative to the implant shoulder. This degree of bone loss aligns with the values typically considered acceptable for long-term implant success [32,33], and this is particularly noteworthy when compared to a recent meta-analysis reporting a mean MBL of 1.90 mm (95% CI: 1.73–2.07) after five years in augmented bone sites treated with standard implants [34]. This suggests that wedge-shaped implants may allow for marginal bone preservation comparable to, or potentially better than, that observed with standard implants placed in augmented bone. Notably, in the present cohort, the mean ridge width was 3.73 ± 0.36 mm—an anatomical condition in which the placement of conventional-diameter implants would typically be contraindicated without associated horizontal augmentation procedures [35,36,37].

Regression analysis identified patient age and implant site as significant predictors of long-term bone resorption, with greater bone loss associated with older age and mandibular placement. The statistically significant association between patient age and marginal bone loss may be explained by the well-documented decline in bone regenerative capacity with aging. Advanced age is associated with reduced osteoblastic activity, delayed vascularization, and an altered inflammatory response, all of which can negatively affect bone remodeling and osseointegration around implants [38,39,40]. These age-related changes may contribute to a less favorable remodeling equilibrium at the peri-implant crestal bone level, particularly in compromised anatomical conditions such as narrow ridges.

In the present study, implant location was significantly associated with marginal bone loss, with mandibular implants exhibiting greater long-term MBL compared to maxillary ones. This finding is consistent with the results of a recent long-term prospective study by Trombelli et al. [41], which reported a significantly higher risk of bone loss ≥2 mm in mandibular implants (OR = 3.00). Although previous studies have shown contrasting results regarding MBL severity in the two jaws [42], these findings highlight the importance of anatomical and biological differences between the maxilla and mandible. Specifically, the mandible is characterized by higher cortical bone density, reduced trabecular content, and limited vascularization—factors that may impair long-term remodeling capacity and increase crestal stress at the bone–implant interface [43,44]. These conditions may be further exacerbated in narrow mandibular ridges, such as those treated in this study, where the residual bone volume is composed almost entirely of cortical bone. In such contexts, the limited presence of spongious bone likely restricts the biological capacity for adaptive remodeling and may contribute to the increased marginal bone loss observed over time. Additionally, an *in vitro* study comparing wedge and conventional implants reported significantly higher buccal bone strain during wedge implant insertion, especially in dense cortical substrates [45]. This mechanical stress may further contribute to the greater marginal bone loss observed in narrow mandibular ridges, where cortical bone predominates. Conversely, smoking status, history of periodontitis, gender, and baseline bone width were not significantly associated with marginal bone loss—though the limited sample size may reduce the power to detect such effects.

Finally, it should be emphasized that the mean marginal bone loss of 1.45 mm at 7 years lies within the thresholds generally considered acceptable for implant success. This indicates that, at the population level, the observed changes are more likely to reflect normal biological remodeling rather than clinically relevant bone modifications. Nevertheless, the relatively high standard deviation (±1.41 mm) indicates considerable inter-individual variability, as illustrated in Figure 2, thereby justifying the exploratory use of regression models to investigate potential associated factors. Thus, while the average MBL is unlikely to have major clinical consequences, understanding the determinants of this variability remains of clinical interest. However, the regression model explained only 17% of the variance in MBL, suggesting that most influencing factors were not captured. Potential confounders such as oral hygiene, occlusal loading, or other patient-related variables were not assessed, which further underlines the exploratory nature of the regression analysis and limits the strength of the conclusions.

No mechanical complications were recorded during the 7-year follow-up, indicating excellent mechanical reliability of both the implant body and prosthetic components. Remarkably, this outcome was achieved despite the reduced bucco-lingual thickness of the implant (1.8 mm), which might raise concerns about structural durability under functional load. The use of high-strength titanium alloy (Grade 5 ELI Ti-6Al-4V) likely contributed to the favorable performance [46,47], along with the implant’s tissue-level configuration. Notably, the thinnest portion of the implant corresponds to a solid, screw-free segment, potentially enhancing resistance to mechanical stress. The absence of prosthetic failures throughout the observation period further supports the long-term feasibility of this design in narrow ridges, where space limitations often require significant structural compromises.

The present study presents several limitations that should be acknowledged. First, the sample size is relatively small, which may reduce the statistical power to detect subtle differences or associations between clinical variables in the multivariate analysis. Nonetheless, the multicenter design—including six independent private clinical practices—and the adoption of broad inclusion criteria enhance external validity by reflecting everyday clinical conditions rather than a single-center research setting. On the other hand, the presence of trained operators for standardized use of piezoelectric preparation and magnetic mallet, together with strict 4-month patient recalls, represent highly controlled conditions that may not be consistently reproducible in routine practice. This combination both strengthens the internal consistency of the data and, at the same time, limits the generalizability of the findings. Second, the absence of a parallel control group treated with conventional implants associated with augmentation procedures in similar anatomical conditions prevents direct comparison and limits conclusions regarding the relative advantages of the wedge-shaped implant design. Future randomized controlled trials with larger cohorts and matched controls are warranted to confirm these findings and more definitively assess the clinical benefits of this minimally invasive approach.

## Figures and Tables

**Table 1:** Demographic and clinical characteristics. SD: standard deviation; F: female, M: male.

Variables	
Age (mean ± SD)	59.6 ± 11.3 years
Gender (F; M) (total 34 patients)	21 (61.8%); 13 (38.2%)
Smoking habits (smoker; no smoker) (total 34 patients)	9 (26.5%); 25 (73.5%)
History of periodontitis (yes; no) (total 34 patients)	14 (41.2%); 20 (58.8%)
Bone crest width at T0 (mean ± SD)	3.73 ± 0.36 mm
Location (mandible; maxilla) (total 45 implants)	33; 12

**Table 2:** Multivariate linear regression using ΔMBL as the dependent variable. F: female; M: male; *: statistically significant.

Variables	Coefficient (β)	Std. Error (Cluster Patient)	95% CI Lower	95% Ci Upper	*p*-Value
Intercept	3.568	2.333	−1.203	8.34	0.137
Age	0.040	0.015	0.009	0.071	0.012 *
Gender (F vs. M)	−0.517	0.470	−1.477	0.443	0.280
Smoking	0.049	0.751	−1.488	1.586	0.948
History of periodontitis	0.326	0.638	−0.979	1.631	0.612
Bone crest width at T0	−0.031	0.566	−1.188	1.126	0.957
Location (mandible)	1.390	0.473	0.419	2.353	0.007 *

## Data Availability

The data presented in this study are available on request from the corresponding author. The data are not publicly available due to privacy and ethical restrictions.
